# Grasp to See—Object Classification Using Flexion Glove with Support Vector Machine

**DOI:** 10.3390/s21041461

**Published:** 2021-02-20

**Authors:** Shun-Hsin Yu, Jen-Shuo Chang, Chia-Hung Dylan Tsai

**Affiliations:** 1 Graduate Degree Program of Robotics, National Yang Ming Chiao Tung University, Hsinchu 30010, Taiwan; sky910011.gdr07g@nctu.edu.tw; 2 Department of Mechanical Engineering, National Yang Ming Chiao Tung University, Hsinchu 30010, Taiwan; kobe.me07g@nctu.edu.tw

**Keywords:** grasping, flex sensing, object classification, machine learning

## Abstract

This paper proposes an object classification method using a flexion glove and machine learning. The classification is performed based on the information obtained from a single grasp on a target object. The flexion glove is developed with five flex sensors mounted on five finger sleeves, and is used for measuring the flexion of individual fingers while grasping an object. Flexion signals are divided into three phases, and they are the phases of picking, holding and releasing, respectively. Grasping features are extracted from the phase of holding for training the support vector machine. Two sets of objects are prepared for the classification test. One is printed-object set and the other is daily-life object set. The printed-object set is for investigating the patterns of grasping with specified shape and size, while the daily-life object set includes nine objects randomly chosen from daily life for demonstrating that the proposed method can be used to identify a wide range of objects. According to the results, the accuracy of the classifications are achieved 95.56% and 88.89% for the sets of printed objects and daily-life objects, respectively. A flexion glove which can perform object classification is successfully developed in this work and is aimed at potential grasp-to-see applications, such as visual impairment aid and recognition in dark space.

## 1. Introduction

Object classification is important and occurs in our daily lives. Typical human classifications rely on three main elements, which are vision, haptic sensing and user experience. In the situation of lacking any of these elements, the classification could become a challenging task. For example, an experienced farmer classifies the quality of crops for grading. The same classification task would be challenging for a rookie, or a vision-impaired operator. Although vision provides fruitful information for object classification, there are scenarios where vision is not available or limited. For example, vision is often not available in a rough terrain due to blockage of lighting during a rescuing mission.

**Figure 1:**
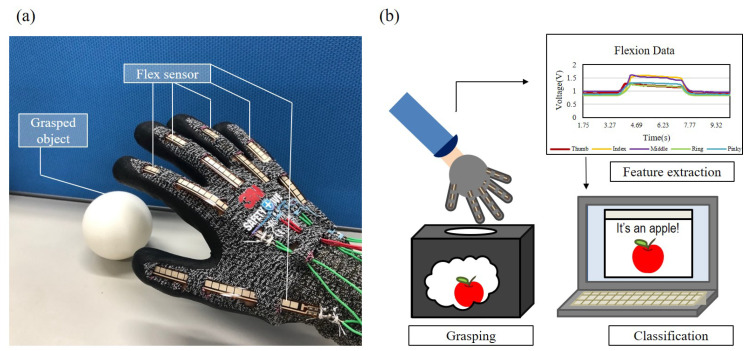
An overview of the proposed flexion glove. (**a**) A photo of the flexion glove. (**b**) The proposed method can classify a target object by a single grasp with the flexion glove.

This paper aims to develop a wearable tool for supporting classification with flexion of fingers, so that classification can become conceivable during an operation, or under the situation that visual recognition is unavailable. Figure 1 shows an overview of the proposed method, where Figure 1a,b are a photo of the proposed flexion glove, and an illustration of the classification process, respectively. The object classification is performed by flexion signals rendered from five flex sensors during a grasp. The flexion, which is also referred as bending in this paper, from each finger is acquired by the flexion glove. Feature values are extracted from the flexion signals and are plugged into support vector machine, a machine learning classifier, for object classification.

Human hand has great potential for object classification because of its special configuration that it has fingers with different lengths and different orientations. Such a configuration results in fruitful information while grasping an object, and also leads to dexterous motion, which robots are difficult to imitate. For example, five fingers of a hand can perform grasping and pinching in coordination with each other [1]. The configuration of human hand is not usually employed in robotic grippers in application because it is complicated and the singularities, in terms of kinematics and controller design, often exist. Even the highly dexterous robots are usually using fingers with the same length. For example, Li et al. developed a method to grasp an object with shape uncertainty, and the hands used in their work have the same length for each finger [2]. In order to take the advantages of human hand configuration on object sensing, a glove for human hands is chosen as the sensing source for our classification method.

Wearable devices for monitoring hand joints motion have been investigated from different perspectives in recent studies [3,4,5,6,7,8,9,10,11,12,13]. For example, Sundaram et al. developed a scalable tactile glove consisting of 548 pressure sensing point over the palm and fingers for learning the signatures of human grasping [5]. Hughes et al. integrate resistive-pressure sensors into a glove for sensing, proprioception and identification [4]. Saggio et al. investigated the electrical features of commercial resistive flex sensors [6]. Sbernini developed a glove using arrays of bend sensors which can measure three joints of a single finger for the posture of a human hand [8]. Such wearable devices can be applied in different fields. For example, sensory gloves have been developed as master-slave inputs for manipulating robot arms [9,10,11]. Wearable devices for rehabilitation purpose were also investigated [14,15,16]. For example, Kang et al. developed a tendon-driven robotic hand for assisting grasping and rehabilitation [17].

Object classification tasks with machine learning are often conducted in the visual aspect. The general operation is to extract the features from the input data, and then perform classifications in the feature space [18,19,20]. While the vision-based hand movement tracking systems are also very popular, they are relatively costly and have limits when the vision of the hand are blocked. Flexion-based sensors have advantages of low-cost, suitable for various of grasping postures, easy to install on a glove, and their long life cycle, which is typically over 1 million times of complete bends. Upon these advantages, the flex-based sensor is chosen for measuring hand gesture in this work.

## 2. Flexion Glove and Classification Method

### 2.1. Development of Flexion Glove

**Figure 2:**
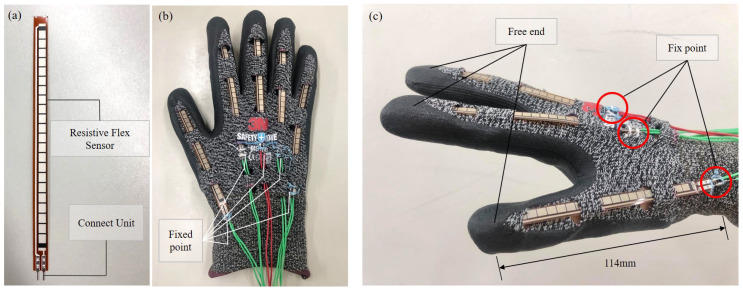
The design of the glove. (**a**) A photo of the flex sensor. (**b**) The arrangement of the sensors on the glove. (**c**) The installment of the sensors.

Figure 2 shows the setup of the glove. Figure 2a is the resistive flex sensor (FS-L-0055-253-ST, spectrasymbol LTD., Salt Lake City, UT, USA) used in this work. The flex sensor measures the amount of bending through the change of the resistance across the sensor. The preliminary test for the sensor calibration was conducted and the results show that the resistance of the sensor is about 15 kΩ without bending, and the resistance can be up to 30 kΩ when the sensor is bend along with the finger which is fully curled.

Figure 2b,c show the photos of flexion glove from top view and side view, respectively. The glove is constructed with five flex sensors mounting on the five fingers sleeves of the glove, as shown in Figure 2b. The length of the flex sensors are 11.4 mm, and are longer than the length of the selves. Thus, the sensors can detect the curling of all finger joints including all proximal interphalangeal (PIP), distal interphalangeal (DIP) and Metacarpophalangeal (MCP) joints. During the sensor installation, the tips of flex sensors and the finger sleeves are first aligned, and the other end of the sensors, the electrodes, are sewn onto the glove, as shown in Figure 2c, for fixing the position of the sensor during the grasping. Since the tips of the sensor and sleeves are not fixed, the sensors can slightly slide during a grasp. One advantage of fixing only one end of the sensor is that the stretching effect of the sensors can be greatly reduced.

**Figure 3:**
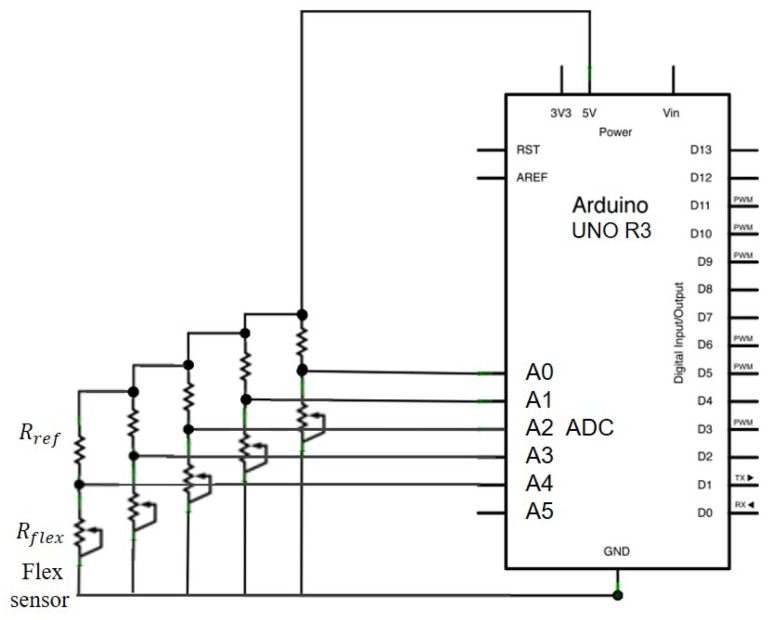
Circuit of flex sensors and microprocessor.

Figure 3 shows the circuit connecting the five flex sensors to a microcontroller (UNO rev3, Arduino Co., Ivrea, Italy). Each flex sensor is represented by an adjustable resistor as $R_{flex}$ shown in the voltage dividers in Figure 3. The signals of bending are acquired from the analog-to-digital converter on the controller. For example, when the bending of the flex sensor results in a resistance value of l$R_{flex}$ = 47 kΩ, the input voltage, 5 V, is divided into half as 2.5 V for the microcontroller. The analog signal will be resolved in 10-bit resolution, that is, 0–5 Volt is divided into 1024 discrete levels. When the resistance goes up as the sensor being bent more, the output voltage would increase accordingly. The resistance of the flex sensor can be calculated from the acquired voltage signal with the following equation

(1) $$R_{flex}=R_{ref}\times \frac{V_{out}}{V_{in}-V_{out}},\;$$

where $R_{flex},R_{ref},V_{in},V_{out}$ are the resistance of the flex sensor, the resistance of the resistor for the voltage divider, the input and output voltage of the voltage divider, respectively. The $V_{min}$ and $V_{max}$ are 0.8 V and 1.8 V, while the input voltage $V_{in}$ is 5 V in this work.

**Figure 4:**
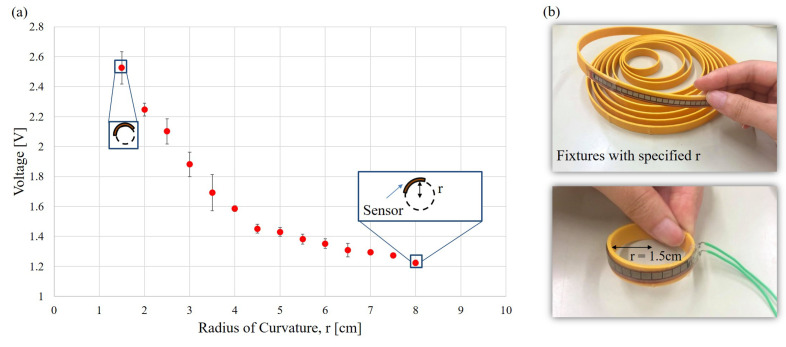
The repeatability test of the flex sensors. (**a**) A photo of the test setup. (**b**) Repeatability results.

Figure 4 shows the repeatability test of the flex sensors. Figure 4a,b show the test results and photos, respectively. Ring-shape fixtures with 14 different radii, r, are printed using a 3D printer. The bending voltage of the flex sensor are measured when the sensor is bent along the fixture surface. The test is repeated 5 times for each fixture. The average values and the standard deviations are plotted, where the ratio between the standard deviation and the voltage signal is 3% in average. The results show that the response of the sensor is fairly stable, particularly for the radii greater than 4 cm.

**Figure 5:**
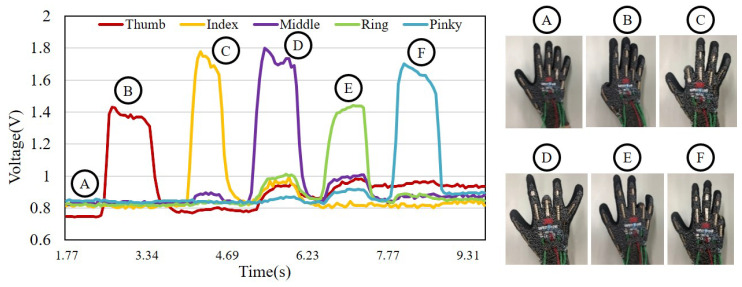
The functional test of the flexion glove. The flexion of each fully curled finger is acquired as voltage signals.

Figure 5 shows the raw data collected from the sensor glove during a functional test. The subject who performed the test is asked to fully curled her fingers one by one and the flexion reflects the change in the voltage signal. Different colors are used to represent the signals of the five fingers in Figure 5. The results show that the bending of each finger can be independently detected.

Hand grasping can be categorized, according to Napier et al. [21], into two types, and they are the power grip and precision grip. Power grip clamps an object firmly by fingers pushing the object against the palm, and has the advantage of a stable grasp. The precision grip pinches an object by the thumb and other fingers without the palm and has a better precision in terms of object handling. In this paper, we focus on power grip which is also the natural approach of human to stably hold an object. Power grip is suitable for object classification because fingers have to adapt to the object’s exterior surface. Classification of precision grip will be tested as a follow-up of this work, and is expected to incorporate with pressure sensors for extracting additional information of target objects.

### 2.2. Classification with Support Vector Machine

Support vector machine (SVM) is a well-known machine learning algorithm for classification [22]. SVM yields great efficiency during the classification because it searches cluster boundaries instead of identifying clusters. The objective of SVM algorithm is to find the hyperplanes with the maximum margin from a class to another in an N-dimensional space, where N is the number of features. The margin of SVM can be expressed as [22].

(2) $$\begin{array}{c} \max_{\omega }\left\{\frac{2}{\left\|\omega \right\|}\right\}\to \min_{\omega }\omega^{T}\omega \\ \mathrm{subject}\;\mathrm{to}\;y_{i}\left(\omega^{T}x+b\right)\geq 1,\forall i=1,\cdots ,n, \end{array}$$

where $\omega ,b,x_{i}$ and $y_{i}$ are the normal vector and the coefficient vector to the hyperplanes, the features of input data set and the corresponding classes, respectively. The distance of the margin which separates two classes is as $\left(2/\|\omega \|\right)$ on the left of Equation (2), and the maximization of it can be represented as the minimization of $\omega^{T}\omega /2$ on the right. Once the training process been done, test cases are categorized based on which side of the hyperplane they fall on for the object classification.

An open-source machine learning library LIBSVM is employed for implementing SVM in python [23]. SVM algorithm, which is a one-verse-one classification, is extended for multiclass classification using a voting algorithm in LIBSVM. The voting algorithm for multiclass is performed by multiple one-verse-one classifications among the classes. If *k* is the number of classes, $C_{2}^{k}=k\left(k-1\right)/2$ one-verse-one classifiers are constructed from all possible combinations of any two classes. The result of each one-verse-one classification is considered a vote, and the class with the highest vote is the classified class.

**Figure 6:**
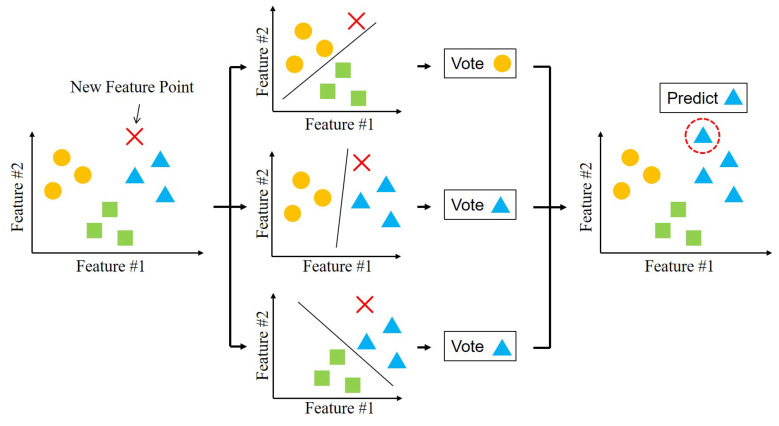
An example of voting strategy for multiclass classification of Support Vector Machine (SVM) in LIBSVM. The marks of circle, triangle and square represent 3 known classes while the cross is a new feature point in the feature space. The cross eventually is classified as triangle class through the voting strategy.

Figure 6 shows an example of the voting algorithm for the classification among three classes. The three classes are plotted as the circle, square and triangle on a feature plane of feature #1 and #2 in Figure 6. A new data point is plotted as the red-crossed mark in Figure 6. The new data set is classified between any two of three classes, as illustrated in the second row in Figure 6. and the determined class gets one vote. Because the class of triangle collects the highest votes among the three classes, the final classification of the new data set will be the triangle class.

## 3. Experiments

### 3.1. Printed Objects and Daily-Life Objects

**Figure 7:**
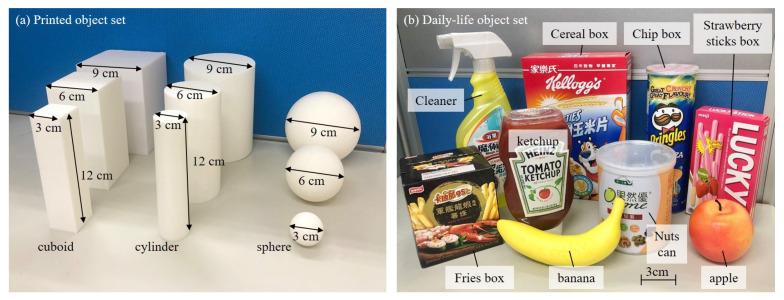
Two sets of objects are tested in the experiments. (**a**) The set of printed objects. (**b**) The set of daily-life objects.

Figure 7 shows photos of the two object sets for evaluating the performance of object classification with the proposed flexion glove. The first object set, as shown in Figure 7a, is named printed object set, which were printed by a 3D printer (K-2327, Kingtec Technical Co., Taipei, Taiwan). The set includes three shapes and each shape has three different sizes. The three shapes of the printed objects, from the left to the right in Figure 7a, are cuboid, cylinder and sphere, while the three different sizes are defined by the shortest distance of the outer-most edges of the cross-section, and are 3 cm, 6 cm and 9 cm, respectively. The shape of cuboid has a squared base, which means the length and width will be the same size. All of the cylinder and cuboid have 12 cm in their heights. The purpose of using printed objects is to explore the effect of shape and size to a grasp, and to determine the reasonable features for the object classification.

The second object set is called daily-life object set, as shown in Figure 7b. The nine objects are randomly chosen and contains a plastic apple, a plastic banana, a cereal box, a cleaner, a chips box, a fries box, a ketchup, a nuts can and a strawberry sticks box. The goal of the test with the daily-life object set is to exhibit the ability of the gloves on recognizing a great variety of objects from daily life.

### 3.2. Experimental Procedures

A step-by-step experimental procedure for object grasping is explained as follows:

1. A volunteer subject is asked to wear the glove and to fully straighten all her fingers for the signal calibrations. The values of the flex sensors are recorded as the initial flexion of the fingers.
2. After the calibration, the subject is asked to perform a two-step operation on the object, including picking up and holding. All objects are placed on the desk one-by-one for the grasping task. The subject is asked to hold the object for 4 seconds before putting it back to its initial position on the desk. The subject is told to freely determine the best grasping strategy for picking and holding the objects.
3. The process is repeated 20 times for one object.

### 3.3. Grasping Phases and Feature Extraction

**Figure 8:**
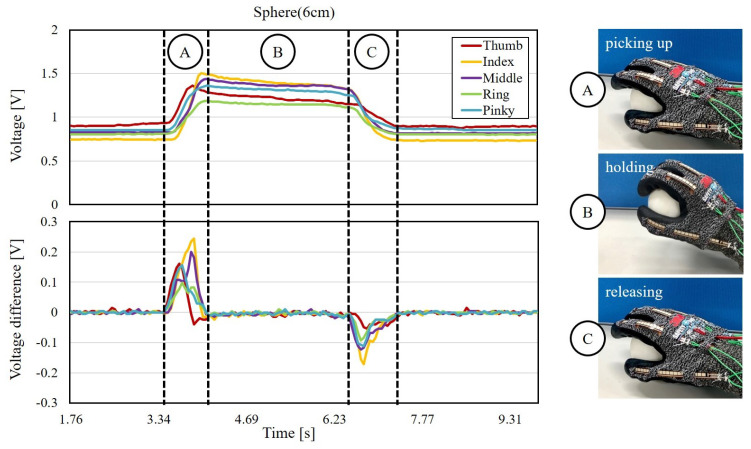
The grasping process is separated into three phases based on the flexion signal.

The flexion signals obtained from the tests were processed for extracting grasping features before applying into the SVM trainer. Figure 8 explains the sensor readouts and its physical meaning with an example of acquired signals from grasping a spherical object with the diameter of 6 cm. The upper and lower charts in Figure 8 are the acquired flexion signals in voltage and the differentiated voltage values with respect to the time, respectively. The signal colors in Figure 8 represent the data from different five fingers. The initial state of the sensor readouts indicates the five fingers are fully straightened at the beginning, and the voltage of the acquired flex signals increases when the finger is curled.

The signals of grasping are separated into three phases, which are picking up, holding and releasing, based on the state of grasping using the differentiated voltage values. The first phase is named the phase of picking up, and is started from the moment when a sudden change of signals is detected from the straightened fingers. This phase is where the subject’s hand is picking up an object, and the fingers are curling up to adapt the exterior of the object in this phase, as shown in Figure 8. The second phase is named as the phase of holding, and is started when the signals are stabilized, meaning the five fingers are maintained in the same posture for holding the object. The phase of holding ends when the signals of sudden declination are detected. The third phase is named the phase of releasing, and is where the five sensors readouts return to the initial states. In this work, all the features are extracted from the phase of holding, where the flexion contains the information of shape and size of the object.

The acquired flexion data from the analog-to-digital converter is collected from five flex sensors and is organized into a matrix *F* as follows:

(3) $$\begin{array}{c} F=\left[\begin{array}{ccccc} f_{1,1} & f_{1,2} & f_{1,3} & f_{1,4} & f_{1,5}\\ f_{2,1} & f_{2,2} & f_{2,3} & f_{2,4} & f_{2,5}\\ \vdots & \vdots & \vdots & \vdots & \vdots \\ f_{M,1} & f_{M,2} & f_{M,3} & f_{M,4} & f_{M,5}, \end{array}\right] \end{array}$$

where $f_{i,j}$ represents the *i*th flexion voltage signal of the *j*th finger in a data sequence, and *M* is the maximum number of collected data. In the test, the sample size *M* and the sampling interval are set as M=300 and Δt=30 ms, respectively. In other words, the five columns of *F* in Equation (3) are the voltages outputs from five fingers. The rows are the sampled data collected from the flex sensors at different moments through data acquisition.

All the three phases have their own physical significance for object classification. In this work, the five flexion signals in the holding phase are chosen as the features for the classification because the fingers are tightly wrapped around the objects, and the flexion of the fingers directly reflects the shape and size of the objects. The holding phase is defined as the region from the peaks to the stable values of sensors readouts, as the holding phase shown in Figure 8. The criterion for determining the stable readouts is defined as follows:

(4) $$f_{i+2,j}-f_{i,j}\leq 0.01\;V,j=1,2,\cdots ,5$$

The first eligible $f_{i,j}$ is the peak of each flexion signal, and it separates the phases of picking up and holding. The signal is defined as stable when the difference in voltages do not exceeded 0.01 V within 60 milliseconds. This criterion has successfully separated all holding phase in 360 data in the tests. The features employed for machine learning here are the averages of flexion signals from all five fingers during the stable period in the phase of holding. The number of objects are 9 for both sets, and thus there will be $C_{2}^{9}=36$ sub-classifications in the SVM for 36 votes to classify a new set of feature points.

## 4. Results and Discussions

The section of results includes three parts, which are the obtained flexion signals on the printed objects, the classification results with SVM, and the results of the “Grasp-to-see” application, respectively. The patterns of flexion signals for grasping objects from the printed set are first investigated. Because the size and shape of the objects are specified, the patterns and trends of flexion signals can be systematically discussed. The second part is focused on the performance of the SVM classifications on the sets of printed objects and daily-life objects. The flexion signals of five fingers are utilized as the input features for SVM. The convergence of the SVM training and the accuracy of the classifications on both object sets are presented. Finally, a real-time application using the proposed flexion glove is demonstrated on daily-life objects. The details of the results are presented in following sections.

### 4.1. Flexion Signals on the Printed Objects

**Figure 9:**
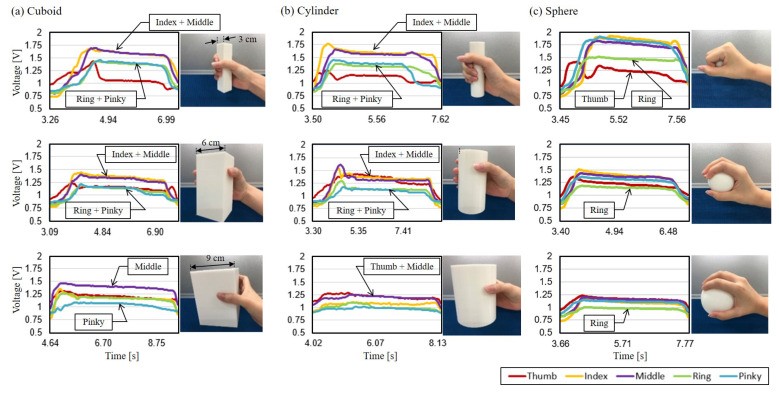
An example of the flexion signals for performing the grasping task on the set of printed objects. (**a**) Cuboid objects. (**b**) Cylinder objects. (**c**) Sphere objects.

Figure 9 shows nine examples of flexion data from grasping each of nine different printed objects, along with grasping photos. For a better visualization of the grasping gestures, the photos in Figure 9 were taken with the same subject performing the grasping without wearing the glove. Figure 9a–c are the flexion results obtained from grasping the objects with the shapes of cuboid, cylinder and sphere, respectively. Each shape has three different sizes as specified in Section 3.1. The grasping of the objects from the smallest to the largest are shown from the top row to the bottom row in Figure 9. Five different colors of curves in each result indicate the flexion of five fingers, where red, yellow, purple, green and blue represent the thumb, index, middle, ring and pinky fingers, respectively.

For grasping cuboid objects, as the examples shown in Figure 9a, the phases of picking, holding and releasing can be clearly distinguished from the figures. As shown in the first two rows of Figure 9a, the index finger and middle finger tend to bend together in the holding phase while the ring finger and the pinky are another group bending together. The flexion of the thumb reaches its peaks during the picking phase and relaxed in the holding phase, except grasping the largest cuboid with the width of 9 cm. Grasping the 9 cm cuboid is a special case with a different pattern of flexion signals that the middle finger bends the most while there is nearly no flexion in the pinky. It could be the result of that the middle finger tried to handle the object with additional flexion in DIP and PIP joints for the largest object.

For grasping cylinder objects, as the examples shown in Figure 9b, the grasping patterns are similar to the ones with cuboids in Figure 9a, except the flexion of the thumb. Two semi-synchronized flexion groups, index-middle and ring-pinky, are bent together in the holding phase for the small and mid-size cylinders. For the largest cylinder, the flexion of the thumb and middle finger are greater than the other three fingers, which can be interpreted as that the thumb and middle finger are dominant in grasping the largest cylinder.

For grasping sphere objects, as the examples shown in Figure 9c, the flexion signals of different fingers tend to be similar, except the one with the smallest sphere where the thumb and ring finger have relatively small flexion comparing with the others. It may be due to that the subject held the sphere at the position of the ring finger in the fist, so that the ring finger cannot curl as much as the index, middle and pinky fingers. For the mid-size and large sphere, all the five fingers have the same tendency which is reasonable as the shape of the objects is fully symmetric.

**Figure 10:**
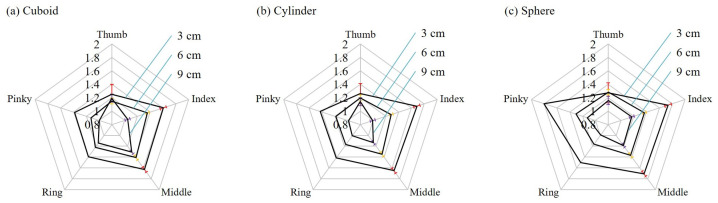
The feature values of each fingers are extracted from the holding phase of a grasping task on the set of printed objects. (**a**) Cuboid objects. (**b**) Cylinder objects. (**c**) Sphere objects.

Figure 10 shows the extracted features from grasping the printed objects. The features are the stable flexion signals during the holding phase. The values are obtained with the criterion in Equation (4) from the signals shown in Figure 9. The averages and standard deviations of the features of all the 180 tests, from 20 repetitions of the same test, are plotted with data points and error bars in the radar plot in Figure 10. A clear tendency can be immediately observed from the three charts in Figure 10 that the flexion signals decrease when the size of the object is increased. It is because when the size of an object increases, the fingers would bend less to fit the exterior of the object. Another observation is that the flexion signal of the thumb usually does not change as much as other fingers while the standard deviations of the thumb are greater than the other four fingers. It can be interpreted as that the subject tends to curl her other four fingers to hold the object, and the role of thumb is dispensable.

According to the results presented in Figure 9 and Figure 10, the patterns of grasping objects of different sizes and shapes are different and have potential to be distinguishable. Therefore, SVM, a machine learning method, is employed for further classification using the flexion signals.

### 4.2. Classification with SVM on Both Object Sets

The grasping task were performed 20 times on the both sets of printed objects and daily-life objects shown in Figure 7a,b, respectively. The features of the flexion signals were extracted with Equations (3) and (4). A total of 360 sets of grasping features were obtained including the 20 times grasping on each of 9 printed objects and 9 daily-life objects.

**Figure 11:**
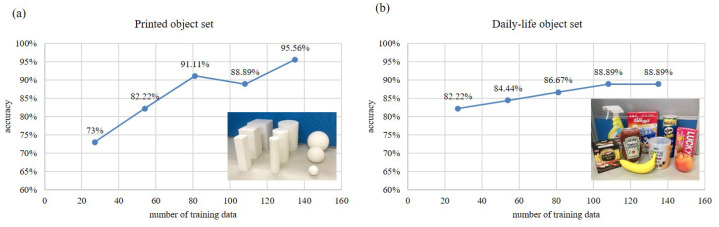
The relation between the number of training data and the accuracy of object classification. (**a**) The results of the set of printed objects. (**b**) The results of the set of dialy-life objects.

Figure 11 shows the convergence test indicating the relation between the number of training data and the accuracy of object classification on the both object sets. For each object set, 45 out of 180 data are utilized for testing the accuracy of the classification while the rest 135 are used for training the classifier. In order to see the trend of the convergence in the training, the numbers of feature sets used to train the classifiers are 27, 54, 81, 108, 135 from the total 135 training sets. The 45 flexion data for testing include 5 times of grasping on 9 different printed objects. Figure 11a shows that with 135 of training data, the accuracy of the classification can be achieved 95.56% in classifying the set of printed objects. Figure 11b shows that with 135 of training data, the accuracy of the classification can be achieved 88.89% in classifying the set of daily-life objects.

**Figure 12:**
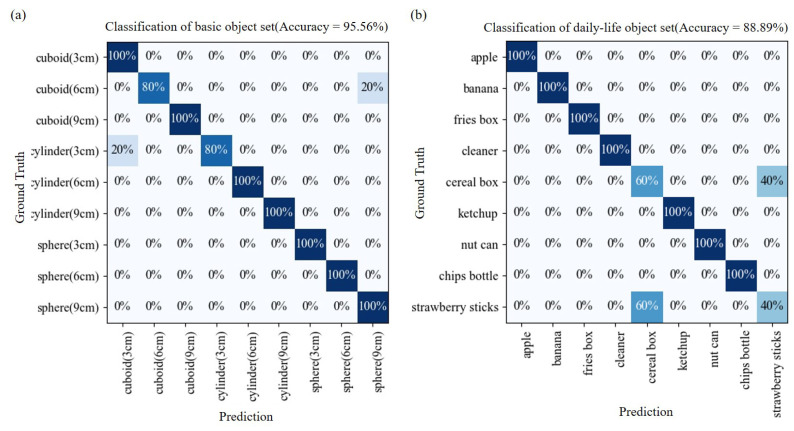
The confusion matrix of classification results on (**a**) printed objects. (**b**) daily-life objects.

A confusion matrix is a common presentation method which is usually used to display the performance of a classification. Figure 12a,b are the confusion matrices for the classification with the set of printed objects and daily-life objects, respectively. Each of the confusion matrix include 45 prediction results. The vertical and horizontal axes of the confusion matrices are the ground truth and the prediction of the SVM classifier. That means, the prediction is more accurate if more data falls in the diagonal, from upper-left to lower-right, of the matrix. The values on the matrix table are the percentages of test data falling in a class. For example, the value of 20% located at the ground truth of “cylinder (3 cm)” and the prediction of “cuboid (3 cm)" in Figure 12a indicates that there is 1 count out of 5 cylinder with the width of 3 cm being misclassified to the category of the 3 cm cuboid. The ideal case for a classification would result in concentrated values along the diagonal of the matrix, which indicate the prediction well match the ground truth.

According to the results in Figure 12a, it is found that the test data almost all fall in the diagonal of the matrix. 43 out of 45 test data is correctly predicted which leads to the accuracy rate of 95.56%. Figure 12b shows the confusion matrix of the predictions on the set of daily-life objects. The nine different objects from the set of daily-life objects have 135 feature sets for training data and 45 for testing, which is the same setup as the set of printed objects. The results show the flexion glove can classify the daily-life objects at the accuracy of 88.89%.

The accuracy of the classify printed object set and daily-life object set are 95.56% and 88.89%, respectively. The reason why the printed object set has higher accuracy could be that the shape and sizes are simple, which make the grasping signals more distinguishable between different classes. While the nine objects from daily-life object set are randomly choose for the grasping task. Some of the shape are more complex, such as the ketchup or cleaner, are the combination of different shapes. The classification also relies on the holding position. Although the subject is not asked to perform the task with a specific holding position, the actual holding positions are quite consistent during the experiments. An additional 15 tasks with a holding position from the top of each printed object were tested, and it was found that the accuracy was reduced from 95% to 86% for classification on the data with the two holding positions. The results suggest that more training data may be needed for dealing with random holding positions.

**Figure 13:**
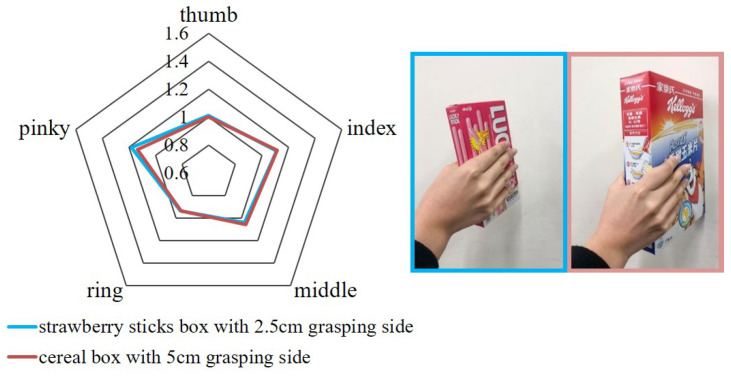
The classes that have been misclassified are putting into radar chart. The radar chart is form by the average of 5 test in each misclassified class.

From the confusion matrix shown in Figure 12b we find that the cereal box and strawberry sticks box are the most frequently misclassified cases among all 9 classes. 40% of cereal box, 2 out of 5, been misclassified as strawberry box and 60% of strawberry box, 3 out of 5, been misclassified as cereal box in the 45 times of test. Figure 13 shows the comparison of the two misclassified cases. It is observed that they all share some extent of similarity in grasping posture regardless of the object shape. For example, in Figure 13 the radar chart is form by the features of holding two misclassified objects, and highly overlapped data are shown. The misclassification is believed due to the reason that the two objects have the similar shape. The size of the sides that were being held were 2.5 cm and 5 cm for strawberry box and cereal box, respectively. The main difference between the posture of holding two objects is the angle between the thumb and index finger while the flexion from the fingers did not change very much. It shows that the glove may not be very sensitive when it comes to the shape of cuboid with different thickness on one side. The success of the classification may be sifnificantly improved with installation of an additional sensor between thumb and index finger.

### 4.3. Grasp-to-See Application

**Figure 14:**
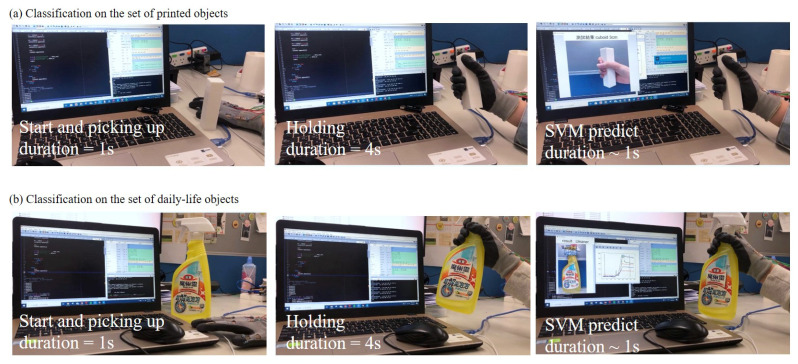
Photos of the grasp-to-see application on the two sets of objects with pre-trained classifiers. (**a**) The set of printed objects. (**b**) The set of daily-life objects.

The glove is able to perform object classification in real-time with pre-trained classifiers. Photos of the real-time tests, which is also known as grasp-to-see application in this paper, are shown in Figure 14, where Figure 14a,b are the step-by-step photos for the classifications on the set of printed objects and the daily-life objects, respectively. In the grasp-to-see application, the user is first asked to sit in front of the computer and to follow the instruction on the screen. Once the test starts, the user is instructed to pick up the object on the desk within 1 s while at the same time, the flexion signals are started being collected through the serial ports on an Arduino board to the SVM program on the computer. After picking up the object, the user is instructed to hold the object for 4 s for the program to extract features from the flexion of his/her five fingers. The extracted features are plugged into the SVM classifier and it takes around 1 to 1.5 s for classifying the object. The whole process takes about 6 s to identify a given objects in the pre-trained classes. It only requires one single grasp for the object classification.

### 4.4. Future Work

The proposed classification, like all other classification methods, has its own limit and chances of misclassifying an object. The method can be further improved by practical solutions. For example, specially designed handles can be made for different tools, so that different types of tools can be better classified for working during the night or in insufficient lighting conditions. Furthermore, the presented results are based on one subject and can also be applied to robotic hands. When the glove is applied to the hands of different subjects, calibration and initial training are needed prior to the test. Initial training is common to different recognition methods, such as fingerprint lock, face lock and so on. The glove is based on supervised learning method and is aimed to perform classification tasks for known objects. A classification glove that can automatically adapt to different subjects for unknown objects is another interesting concept, and can be a follow-up work to the proposed glove.

## 5. Conclusions

The proposed flexion glove achieves feature extraction and object classification through flexion sensing and machine learning classifier. The glove is expected to assist object recognition in the situations of the user lack of experience in classify different classes of objects or insufficient light condition. The real-time classification system enables users to obtain meaningful flexion information in a single grasp. In the experiments, the 360 sets of flexion signals from grasping two different sets of objects are employed for both SVM training and testing. The accuracy of 95.56% is obtained with the set of printed objects using 45 sets of test data after 135 sets of training data, while the accuracy of 88.89% is obtained with the set of daily-life objects using the same numbers of training and testing. The results demonstrate the feasibility of object classification using the developed flexion glove. The glove could be a low-cost solution for the applications for grading agricultural crops, identification in low-lighting environments and other grasp-to-see applications.
